# Life and Death of Activated T Cells: How Are They Different from Naïve T Cells?

**DOI:** 10.3389/fimmu.2017.01809

**Published:** 2017-12-13

**Authors:** Yifan Zhan, Emma M. Carrington, Yuxia Zhang, Susanne Heinzel, Andrew M. Lew

**Affiliations:** ^1^The Walter and Eliza Hall Institute of Medical Research, Parkville, VIC, Australia; ^2^Department of Medical Biology, University of Melbourne, Parkville, VIC, Australia; ^3^Guangzhou Institute of Paediatrics, Guangzhou Women and Children’s Medical Centre, Guangzhou Medical University, Guangzhou, Guangdong, China; ^4^Department of Microbiology and Immunology, Peter Doherty Institute for Infection and Immunity, University of Melbourne, Parkville, VIC, Australia

**Keywords:** activated T cells, apoptosis, necroptosis, epigenetics, autoimmunity, treatment

## Abstract

T cells are pivotal in immunity and immunopathology. After activation, T cells undergo a clonal expansion and differentiation followed by a contraction phase, once the pathogen has been cleared. Cell survival and cell death are critical for controlling the numbers of naïve T cells, effector, and memory T cells. While naïve T cell survival has been studied for a long time, more effort has gone into understanding the survival and death of activated T cells. Despite this effort, there is still much to be learnt about T cell survival, as T cells transition from naïve to effector to memory. One key advance is the development of inhibitors that may allow the temporal study of survival mechanisms operating in these distinct cell states. Naïve T cells were highly reliant on BCL-2 and sensitive to BCL-2 inhibition. Activated T cells are remarkably different in their regulation of apoptosis by pro- and antiapoptotic members of the BCL-2 family, rendering them differentially sensitive to antagonists blocking the function of one or more members of this family. Recent progress in understanding other programmed cell death mechanisms, especially necroptosis, suggests a unique role for alternative pathways in regulating death of activated T cells. Furthermore, we highlight a mechanism of epigenetic regulation of cell survival unique to activated T cells. Together, we present an update of our current understanding of the survival requirement of activated T cells.

## Introduction

Death is fundamental to cellular development and response, with immune cells no exception. It facilitates the selection and retention of desirable clonotypes, while ridding the population of superfluous or often harmful cells. As T cells only become functional upon activation, understanding the control and transition of survival mechanisms in naive, activated and memory T cells is crucial to our ability to harness T cell responses or limit pathology in situations where destructive T cells survive. Although the control of naïve T cell survival has been mostly resolved, how activated T cells regulate their survival is less well understood, despite thorough investigation. In this review, the contribution of several cell death pathways to the life and death of activated T cells will be discussed.

T cells consist of many subtypes including TCRαβ-bearing conventional T cells, Treg cells, TCRγδ T cells, and T cells expressing invariant or semi-invariant TCR chains (such as NKT cells and MAIT cells). Most of our current understanding of T cell survival has largely been focused on two types of TCRαβ-bearing T cells: conventional CD4^+^ and CD8^+^ T cells. After development in the thymus, conventional T cells exist in naïve form. Upon activation, they undergo clonal expansion and gain different effector functions. A small fraction of activated cells become long-lived memory cells. It has been appreciated for a long time that naïve T cells and activated T cells differ in their survival program (1, 2). In this section, we will discuss the findings from recent studies investigating the role of pro- and antiapoptotic molecules in activated T cells by analysis of their expression patterns, the use of selective inhibitors, and the genetic deletion of genes in these cell death pathways. The use of selective antagonists offers several advantages. First, they allow quantitative dissection of the contributions of individual antiapoptotic molecules. Second, they allow us to separate the developmental or precursor effects from direct effects in the activated cells. The inhibitor approach is particularly useful for the *in vitro* dissection of survival requirements of T cells. This approach can also be used for dissection of *in vivo* survival requirement of T cells. However, the *in vivo* application can be complicated by effects of antagonists on cells other than T cells, which in turn influence T cell survival. Third, and perhaps most importantly, they may have the therapeutic potential for curtailing unwanted T-cell responses.

## BCL-2 Intrinsic Pathway of Apoptosis

**Figure 1:**
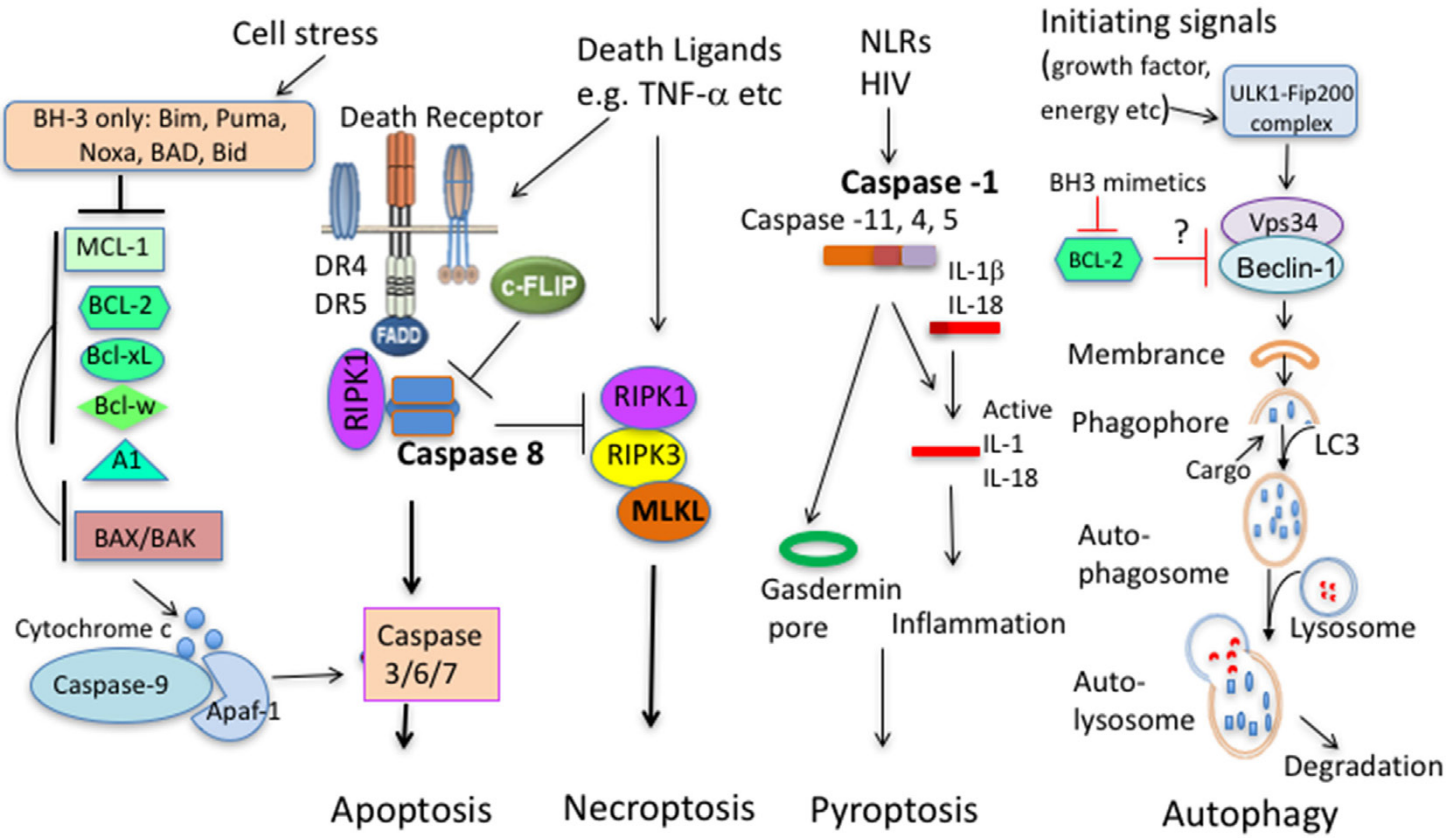
Principal pathways of cell death. Apoptosis comprises of the intrinsic and extrinsic pathway. In the intrinsic pathway, cells sense stress signals, leading to upregulation and activation of BH3 proteins. When antiapoptotic molecules that normally bind and keep BH3 proteins and/or BAX/BAK in check are displaced, BH3 proteins will trigger activation of BAX and BAK. BAX/BAK then mediate cytochrome *c* release from the mitochondrial outer membrane to the cytosol, activating Caspase-9 and downstream caspases leading to cell demise. In the extrinsic pathway, extracellular ligands engage cell death receptors, leading to formation of the death-inducing signaling complex (DISC) with the adaptor protein Fas-associated death domain protein (FADD) and pro-caspase 8, leading to activation of caspase 8 and subsequent activation of effector caspases and apoptosis. In this pathway, c-FLIP acts as a negative regulator. c-FLIP is structurally highly similar to procaspase-8 but lacks catalytic activity, thus outcompetes caspase 8 binding blunting the death-inducing signal. When extrinsic apoptosis in inhibited (Caspase 8 deficiency, caspase inhibition, and high c-FLIP expression), engagement of death ligand can initiate necroptosis that involves activation of the necroptosome comprising RIPK1, RIPK3, and mixed lineage kinase domain-like (MLKL). Pytoptosis is a type of cell death initiated from activation of several Caspases that cleave IL-1β and IL-18. A downstream molecule Gasdermin is critical for cell death by pyroptosis. Autophagy promotes proteolytic degradation of mitochondria and other cytosolic components at the lysosome. It can promote survival or diminish survival depending on degraded molecules. BCL-2 family members with antiapoptotic and proapoptotic molecules can interact with upstream autophagy signaling molecules.

The BCL-2 family can be separated into three groups, the pro-survival molecules BCL-2, BCL-XL, BCL-W, MCL-1, and A1/BFL1; the group of BH3-only pro-apoptotic molecules BID, BIM, PUMA/BBC3, BAD, NOXA/PMAIP, BIK/BLK/NBK, BMF, and HRK/DP5; and the pro-apoptotic “effectors” BAX and BAK (3) (Figure 1). The interplay of these molecules is a finely orchestrated system. As antiapoptotic proteins sequester BH3 proteins that initiate apoptosis, BH3 proteins require BAX/BAK for apoptosis induction as multiple BH3 proteins fail to induce apoptosis in BAX^−/−^/BAK^−/−^ system while reintroduction of BAX restores the ability of BH3 proteins to induce apoptosis (4, 5). When BH3 protein function becomes dominant, the pro-apoptotic “effectors” proteins BAX and BAK will permeabilize the mitochondrial outer membrane, leading to cytochrome *c* release into the cytosol to assemble with APAF-1 and pro-caspase 9 to form the apoptosome, followed by the activation of effector caspases. Our most recent studies suggest that immune cell survival is controlled by the quantitative participation of multiple antiapoptotic proteins (6). Nevertheless, their contribution to T cell survival is not equal, probably related to their dynamic regulation of expression and lifespan. Below we will discuss the BCL-2 antiapoptotic molecules separately.

### BCL-2

BCL-2 is the prototype of BCL-2 family members and has been the most extensively studied. Overexpression of BCL-2 delays T-cell death (7, 8) while BCL-2 deficiency reduced T-cell survival (9, 10). Survival of naïve T cells mediated by BCL-2 was largely dependent on IL-7 as BCL-2 rescued the severe defect in T cells in IL-7R-deficient mice (11, 12). Naive T cells almost exclusively express BCL-2 and are heavily dependent on BCL-2 for survival since they show high sensitivity to BCL-2 antagonist ABT-199 (6). Similar findings have also been derived from earlier studies with ABT-737, an inhibitor with a broader binding activity to BCL-2, BCL-xL, and BCL-w (13–17). It is evident from these studies that activated T cells including effector and memory T cells are less sensitive to the inhibitor when compared to naïve T cells. However, there are some variations in sensitivity between activated CD4^+^ and CD8^+^ T cells. Compared to CD4^+^ T cells, CD8^+^ memory T cells are relatively sensitive to BCL-2 inhibition with ABT-737 (14–16). However, ABT-737 killed only a relatively minor fraction of CD8^+^ OT-1 T cells under optimal stimulation *in vitro* (18). As for CD4^+^ T cells, it is also revealed that Th1 memory cells are long-lived while Th17 cells are short-lived, probably related to their lower expression of BCL-2 (19). It remains to be determined whether functionally different T cells show differential survival requirements for BCL-2. A simple interpretation regarding different sensitivity to BCL-2 antagonist by naïve and activated T cells is that T cells alter their survival program when T cells get activated. They downregulate their BCL-2 sensitivity while at the same time upregulate A1, BCL-xL and MCL-1 (18, 20, 21). This in turn may lead to a higher resistance to BCL-2 inhibitors *in vitro* and *in vivo*.

### BCL-xL and BCL-w

It had been shown that genetic elimination of BCL-xL in the mouse reduces the survival of double-positive thymocytes but not the survival of single-positive thymocytes in culture or peripheral T cells *in vivo* (22). Early studies demonstrated that BCL-xL was an activation-upregulated antiapoptotic molecule promoting survival of activated T cells and memory T cells (23, 24); however, more recent studies have shown that BCL-xL is dispensable for the generation of effector and memory T cells (25). Recently, highly selective BCL-xL inhibitors have been developed (26), but have shown minimal T-cell killing ability (unpublished). BCL-w is less studied, although it can be expressed by some subsets of T cells (27). The similarity of action on T cells between ABT-737 (antagonizing BCL-2, BCL-xL, and BCL-w) and the BCL-2 specific ABT-199 suggests that BCL-w has little impact on T cell survival (6, 15). No T cell defects have been documented in BCL-w-deficient mice (28).

### MCL-1

As global deletion of MCL-1 in mice led to embryonic lethality (29), conditional deletion of MCL-1 has been adopted to examine its contribution to T-cell development and survival (30). It has been shown that the development and maintenance of T cells requires MCL-1, implying that MCL-1 is also important for the survival of naïve T cells (30). These findings are also supported by the characterization of mice with MCL-1 haplodeficiency (6). Conversely, overexpression of MCL-1 can promote T cell development and survival (6, 31). Even for developing T cells, MCL-1 seems to have a unique role in supporting T cell development, as the developmental defects arising from deletion of MCL-1 can be partially rescued by BAK deficiency but not by BAX deficiency or overexpression of BCL-2 (32). During activation, MCL-1, along with A1 and BCL-xL show elevated expression in T cells (18, 21). In a system of inducible deletion of MCL-1, Mx1Cre-induced deletion of Mcl-1 led to massive loss of antigen-specific T cells in LCMV-infected mice (21). Notably, loss of activated T cells with MCL-1 deletion could be rescued with concomitant loss of BAX and BAK but not BIM (21). In contrast, BIM deficiency could rescue T cell defect caused by BCL-2 deficiency (13). Considering that activated T cells expressed not only BIM but also PUMA and NOXA (21), perhaps multiple BH3 proteins participate to regulate cell death. At least *in vitro*, stimulated human T cells seem to depend on the MCL-1/NOXA axis for survival (33). The recent development of the selective MCL-1 inhibitor (34) also allows timely dissection of MCL-1 contribution to T cell death *in vitro* (6). It could induce significant death of T cells (6). Together with revelation of the importance of MCL-1 in regulating Treg cell survival (35), MCL-1 is a key antiapoptotic molecule for T cell survival.

### A1

A1 was initially identified as a GM-CSF regulated pro-survival gene and its expression restricted to the hematopoietic system (36). In T cells, A1 is largely only induced upon TCR stimulation (18, 37). Several studies have demonstrated an association of A1 upregulation with enhanced T cell survival (38, 39). Due to the quadruplication of *A1* genes in mice, mice with a full deletion of *A1* genes only recently became available, allowing for a direct assessment of A1’s contribution to T cell survival. Somewhat surprisingly, A1 deficiency has a relatively minor impact on T-cell survival and the induction of T-cell response (40, 41). Nevertheless, when other antiapoptotic molecules were suppressed by inhibitors, A1 deficiency could result in significantly poorer survival of T cells (6).

### Pro-Apoptotic BH3 Proteins and T Cell Survival

BIM has been shown to have a dominant role in regulation of T cell survival among the BH3 proteins (13, 42). The deletion of immature autoreactive thymocytes was defective in BIM-deficient mice, leading accumulation of T cells with self-reactivity (43, 44). For mature T cells reaching the periphery, BIM is also important for survival of naïve T cells (45), which was found to be partly regulated *via* IL-7 signaling (46, 47). Upon activation, BIM is also crucial for the termination of T-cell immune response against acute infection with herpes simplex virus (48). Similarly, peripheral deletion of activated T cells is also mediated by BIM (49, 50). Interestingly, during a chronic infection, activated T cells with different antigen specificity showed differential requirements from BIM (51). It is proposed that infection duration and antigen loads may switch apoptosis pathways for activated T cells (51). Apart from BIM, other BH3 proteins also have a non-redundant role in regulating survival of activated T cells. Deficiency in NOXA could lead to accumulation of activated T cells and immunopathology during chronic LCMV infection (52, 53). Interestingly, PUMA but not NOXA, BID, or BAD was shown to have a non-redundant role in protection from cell death of antigen-specific T cells in HSV-1 infection (54). The basis of the varied requirement for different BH3 proteins remains unclear. Furthermore, BH3 proteins can collaborate to regulate T-cell survival. It has been reported that the combined loss of PUMA and BIM protected mitogen-induced T cell blasts from IL-2 deprivation-induced death more potently than the loss of BIM *in vitro* (55). BIM also collaborates with NOXA or PUMA to control effector CD8^+^ T-cell responses during CMV infection, probably by targeting different antiapoptotic molecules (56). Even without infection, simultaneous defects in both BIM and PUMA could lead to severe forms of autoimmunity and organ damage (57). BIM also cooperated with BID for contraction of the anti-viral T cell response (58). Furthermore, three concurrent studies also demonstrated that BIM and Fas, a key molecule of extrinsic apoptosis pathway, also cooperate to regulate different types of T-cell responses (59–61). Thus, BH3 proteins, particularly BIM, are key molecules to limit T-cell survival.

### BAK/BAX and T Cell Survival

Multidomain pro-apoptotic molecules BAK/BAX are often referred as “effectors” of the intrinsic mitochondrial cell death pathway and are essential, yet each individually redundant, for T-cell apoptosis (62). As individual BH3, only proteins may be redundant and could not rescue the T cell defect caused by MCL-1 deficiency, concomitant loss of BAX and BAK rescued the loss of activated T cells with MCL-1 deletion (21). Thus, BAX and BAK are critical for apoptosis of activated T cells and naïve T cells. However, careful examination has revealed subtle differences between BAK and BAX in apoptosis induction. BAK binds preferentially to MCL-1 and BCL-xL (63). Fittingly, loss of BAK was able to partially rescue T-cell defects caused by conditional deletion of MCL-1, whereas overexpression of BCL-2 or loss of BAX was unable to rescue the cell (32). Notably, chimeric mice reconstituted with BAK^−/−^ bone marrow cells, but not BAX^−/−^ bone marrow cells, developed immunopathology and died prematurely (64). In humans, mutations in BAK have been associated with some forms of autoimmune disease (65). Thus, this group of pro-apoptotic molecules is also dynamically involved in regulation of T-cell survival.

## Other Mechanisms of T Cell Death

There is great interest to repurpose BCL-2 antagonists that have been approved for cancer treatment to dampen inflammation (66–68). Notably, for T cell-mediated inflammation (collagen-induced arthritis), ABT-737 (antagonizing BCL-2, BCL-xL, and BCL-w) was only effective before but not after induction of disease (67). Differential sensitivity to ABT-737 by naïve T cells and activated T cells may offer an explanation to above discrepancy. Given that a large fraction of activated T cells can still survive even when all antiapoptotic molecules were impaired (6), other mechanisms must contribute to the survival of activated T cells. Here, we provide a brief summary what impact other death pathways have on T-cell survival. Many of these pathways are inter-connected, resulting in a complicated regulatory network balancing T-cell-mediated immunity and tolerance.

### The Extrinsic Pathway of Apoptosis

Soon after BCL-2 was discovered as a key player of the intrinsic pathway of apoptosis (69), Fas/FasL were discovered as the prototype receptor/ligand pair of the extrinsic (death receptor) pathway of apoptosis (70, 71). Fas, upon engagement to FasL, forms the death-inducing signaling complex with the adaptor protein Fas-associated death domain protein and pro-caspase 8, leading to activation of caspase 8 and subsequent activation of effector caspases and apoptosis (72). Deletion of Fas and FasL in mice resulted in lymphadenopathy and an increase in the unusual TCRαβ^+^B220^+^ CD4^−^CD8^−^ (DN) T cells (73, 74). Human mutations in CD95 also resulted in increased TCRαβ^+^B220^+^ CD4^−^CD8^−^ DN T cells and were associated with the development of autoimmune lymphoproliferative syndrome (75). Thus, the death receptor apoptotic pathway is important for T cell homeostasis.

However, the importance of this pathway in clearance of activated T cells seems to be dependent on the experimental conditions [reviewed in Ref. (42)]. Early on, *in vitro* induction of activation-induced cell death (AICD) in T cells has been found to be critically dependent on Fas and FasL interaction (76–78). *In vivo*, deletion of SEB-activated T cells in mice was impaired with defective FasL–Fas pathway in some (79, 80) but not in other studies (49). Similarly, deletion of antigen-activated CD8^+^ T cells during acute a viral (HSV-1) infection was not affected by Fas deficiency (48, 81) but was impaired during a persistent chronic infection (82). Similarly, work on c-FLIP_L_ in T cells, a classical negative regulator of death receptor/extrinsic pathway signaling, also generated controversial results. c-FLIP_L_-deficient T cells were shown to display enhanced cell death upon TCR stimulation (83), while an earlier study found that activation-induced death of T cells in c-FLIP_L_ transgenic mice was unaffected (84). The nature of an immune response—acute *vs* chronic infection, transient *vs* repeat stimulation or signal strength of T cell activation has been offered as potential explanations for the reported varied dependency (42, 61).

There are two other complex aspects regarding the pathway in regulation of T cell survival. First, AICD could occur *via* the interaction of death receptor and their ligands other than Fas/FasL. TNF-α/TNF receptor 1 and TRAIL/DR4/DR5 also contribute to AICD (85, 86). For TNF-mediated AICD, it has been reported that soluble TNF-α but not transmembrane TNF-α (tmTNF-α) induced AICD *in vitro* and *in vivo* (87). A more recent study showed that tmTNF-α could promote AICD *via* reverse signaling in which tmTNF-α behaves as a receptor to interact TNFR (88). Furthermore, the ligand binding to death receptor results in not only downstream activation of initiator caspases 8 and 10 (89) but also of prosurvival signaling pathways, including nuclear factor-κB and mitogen-activated protein kinase (89). It remains to be fully appreciated how a final outcome (death *vs* life) is determined when a ligand binds death receptor.

Second, apart from cooperating with the intrinsic apoptosis pathway to regulate T-cell death (59–61), extrinsic apoptosis pathway is also heavily entangled with programed necroptosis. Despite caspase activity is critical for death receptor-mediated apoptosis, Fas can trigger Caspase-8-independent death of activated human and murine T cells (90, 91). The death pathway (necroptosis) involves the receptor-interacting serine-threonine kinases RIPK 1 and RIPK3 (90, 91) (Figure 1). The involvement of necroptosis in T cell death is discussed in the following section.

### Necroptosis

Necroptosis requires activation of signaling complex consisting of RIPK1, RIPK3, and mixed lineage kinase domain-like (MLKL). When activity of Caspase 8 is absent or suppressed, three key components will assemble the necroptosome (Figure 1). Both RIPK1 and RIPK3 are key upstream components of TNF signaling and can mediate apoptosis, necroptosis, and inflammation while MLKL, as a downstream signaling molecules is primarily involved in necroptosis (92). At least *in vitro*, TNF can induce necroptosis in the absence of RIPK1 (93). As for activated T cells, two aforementioned studies demonstrated that necroptosis can occur at certain conditions (90, 91). For death ligand-mediated necrotptosis, RIPK1 is essential for cell death (90). For TCR-mediated necrotptosis (at least without exogenous death ligands), necroptosis occurred in the absence of Caspase 8 is rescued by RIPK3 deficiency and partially rescued by RIPK1 inhibition with necrostatin-1 (91). Notably, necroptosis occurred *in vitro* in actively proliferating cells (91). *In vivo*, RIP3 deficiency only prevent the loss of Caspase 8^−/−^ T cells in expansion phase but not in contraction phase during a viral infection (91), implying a stage-specific role. MLKL has been identified as a key player in necroptosis of fibroblasts and macrophages triggered by TNF in conjunction with caspase inhibitors and IAP inhibitors (94, 95). Notably, T cells develop normally in MLKL knockout mice (95, 96). As most myeloid cells constitutively express high levels of MLKL, naïve T cells express low levels of MLKL, but display an increase in MLKL expression upon activation (97). Currently, the importance of MLKL in regulating the death of activated T cells is unknown. Overall, necroptosis of activated T cells is mostly prominent when caspase activity is suppressed. Significance of the pathway in regulation of T cell survival remains to be established.

### Pyroptosis

Pyroptosis describes pro-inflammatory programmed cell death (98). Differing from classical apoptosis, pyroptosis employs inflammatory pyroptotic caspases (caspase-1, -4, -5, -11). Caspase-1-dependent and inflammation-induced pyroptosis is critical for CD4 T-cell death in HIV-infected host (99). It is unclear whether pyroptosis is involved in the death of TCR-triggered T cells.

### Autophagy

Autophagy promotes proteolytic degradation of mitochondria and other cytosolic components at the lysosome. It can promote or diminish cell survival depending on degraded molecules. We mainly discuss here the role of autophagy in regulation of T cell survival, although autophagy can influence the induction and maintenance of an immune response independent of cell survival mechanisms (100). Several reports showed that autophagy promotes T-cell survival (101–103). *In vitro* dissection of contribution of autophagy to AICD had demonstrated that both the activity of upstream kinase AMPK and key downstream molecule LC3 in autophagy signaling was reduced upon AICD induction, leading to the accumulation of damaged mitochondrial and apoptosis progression. T cells from mouse models defective in autophagy had higher sensitivity to AICD (104). Apart from inhibition of autophagy leading to accumulation of damaged mitochondrial, TCR signaling during AICD induction can also lead to mitochondrial fragmentation in a Drp1-dependent fashion, resulting in AICD involving reactive oxygen species and CD95 induction (105). Beyond the potential contribution of autophagy to regulate cell survival at early activation stage, both CD4^+^ and CD8^+^ memory T cells have been shown to be preferentially affected by the autophagy process (106, 107). Nevertheless, how this pathway impacts on survival of activated T cells remains to be fully explored. Particularly, how does this pathway interplay with BCL-2-regulated apoptosis? It has been reported that autophagy enhances degradation of pro-apoptotic proteins such as BIM and various caspases (103). Upstream of autophagy signaling, interaction of antiapoptotic molecules and pro-apoptotic molecules with key signaling molecules of autophagy remains controversial. Beclin-1 is a BCL-2-binding protein that is essential to autophagy (108). BCL-2 can inhibit Bectin-1-dependent autophagy to maintain autophagy at levels that are compatible with cell survival (109). However, a more recent study showed that BCL-2 or other antiapoptotic molecules do not directly inhibit components of the autophagic pathway but affect autophagy indirectly by inhibition of Bax/Bak (110). The findings are in contrast to a subsequent study showing that the longer exposure of BH3 mimetic ABT-737 induces autophagy through a BAX and BAK-independent mechanism (111). In addition, it has been shown that BIM directly interacts with Beclin-1 to inhibit autophagy (112). It remains to be investigated how these players in BCL-2-regulated pathways affect autophagy of T cells and the consequences of these effects.

### Epigenetic Control of Activated T Cell Survival

**Figure 2:**
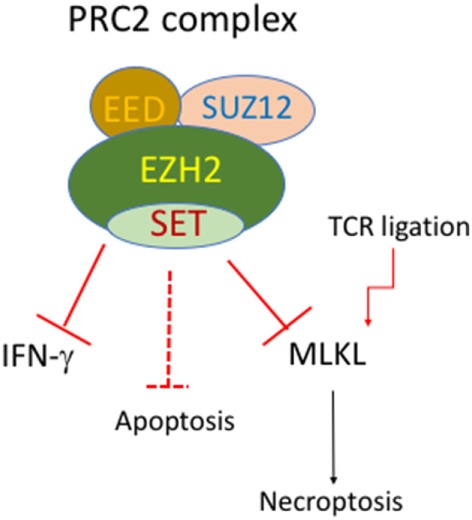
Putative roles of EZH2 and PRC2 complex in regulation of cell death of activated T cells. The polycomb repressive complex (PRC) contains EZH1/2, SUZ12, and EED. EZH2 deficiency in T-cell lineage does not affect normal development of CD4 and CD8 T cells. However, EZH2-deficient T cells display enhanced Th1 differentiation and enhanced cell death upon activation. EZH2-deficient T cells also have enhanced mixed lineage kinase domain-like (MLKL) expression upon TCR stimulation.

Epigenetic regulation through DNA methylation and histone modification is essential to fine-tune gene expression. Depletion of the methyltransferase SUV39H1, which mediates H3K9 trimethylation in Th2 cells, can lead to the transcription of Th1 cytokine IFN-γ (113). EZH2 (Enhancer of zeste homolog 2) is another histone methyltransferase that catalyzes H3K27me3 and acts primarily as a gene silencer. EZH2 is a key component of the polycomb repressive complex (PRC) 2, which also contains SUZ12 and EED (Figure 2). It has emerged that EZH2 is critically involved in regulation of cell survival and differentiation of activated T cells. T-cell lineage specific deletion of EZH2 (CD4-Cre/EZH2^fl/fl^) did not alter normal development of CD4 and CD8 T cells. However, it led to enhanced Th1 and Th2 differentiation (114). Counterintuitively, suppression or loss of EZH2 also accelerated the death of activated T cells (97, 115, 116). The precise mechanisms of cell death that cause loss of activated T cells are currently unknown. Expression of both antiapoptotic and pro-apoptotic molecules were higher in activated EZH2^−/−^ T cells, compared to activated WT T cells (116). Notably, deletion of Bim did not prevent the loss of activated EZH2^−/−^ T cells (116). Somewhat surprisingly, despite massive loss of GVDH causing T cells, antileukemia T cells of EZH2^−/−^ donor origin were preserved. Similar to the example of differential requirements for BIM for T cells with different antigen specificity (51), stimulation duration and strength may cause differential dependence on EZH2 for survival. In addition, we found that the induction of MLKL in activated T cells was enhanced by EZH2 deletion (97). This raises the possibility that MLKL-mediated necroptosis contributes to loss of activated T cells in EZH2-deficient mice. It has also been shown that loss of EZH2 in donor T cells has been shown to inhibit GVHD in mice after allogeneic bone marrow transplantation (115, 116), indicating that manipulation of PRC2 signaling may offer an avenue to specifically target activated cells.

## Concluding Remarks

T cells are a key component of the immune system and play a critical role in orchestrating the immune responses to self and foreign antigens. The magnitude of the T-cell response is critically regulated by cell survival/death. Conceivably, targeting the survival mechanisms may provide an avenue for immune intervention. Enhancement of T cell survival can be beneficial in situations of immune deficiency, immunization, and cancer immunotherapy. On the other hand, an inappropriate immune response (e.g., autoimmunity and transplant rejection) can be curtailed by inducing T-cell death. We now appreciate that survival control of naïve and activated T cells is different and that multiple pathways contribute to survival control of activated T cells (Table 1). Currently, there are still many unknowns regarding how life and death of activated T cells is regulated. A better understanding of how the survival of T cells, particularly activated T cells, is regulated should increase the potential to harnessing T-cell immune responses.

**Table 1:** Survival requirement of T cells at a glance.

Death pathways	Naïve T cells	Activated T cells	
BCL-2 regulated apoptosis (intrinsic)	BCL-2++++ MCl-1++ High expression of BCL-2	MCL-1+++ A1+ BCL-2++ (CD8 memory) High expression of MCL-1, A1, BCL-xL	

	Bim+++	Bim+++ Puma+, NOXA+, BID+ (prominent at contraction phase)	

Death receptor-regulated apoptosis (extrinsic)	+/−	++ (prominent at persistent antigen stimulation)	

Necroptosis	−/+	++ (when Caspase 8 disabled) (mainly expansion phase)	
	Low mixed lineage kinase domain-like (MLKL) expression	High MLKL expression	

Pyroptosis	+/−	HIV-infected CD4	

Autophagy	+/−	++	+++

*++++ > +++ > ++ > + >—illustrating the degree of dependence by T cells on the indicated molecules or pathways*.

## Author Contributions

All authors listed have made a substantial, direct, and intellectual contribution to the work and approved it for publication.

## Conflict of Interest Statement

The authors declare that the research was conducted in the absence of any commercial or financial relationships that could be construed as a potential conflict of interest.
